# Successful use of rivaroxaban achieving therapeutic anti-factor xa levels in a morbidly obese patient with acute intermediate-high risk pulmonary embolism

**DOI:** 10.1590/1677-5449.202300562

**Published:** 2023-06-30

**Authors:** Kingsley Dah, Mateo Porres-Aguilar, Alan De la Rosa, Swathi Prakash

**Affiliations:** 1 Texas Tech University Health Sciences Center El Paso, Paul L. Foster School of Medicine, El Paso, Texas, USA.

**Keywords:** direct oral anticoagulants, rivaroxaban, venous thromboembolism, morbid obesity, acute pulmonary embolism, anticoagulantes orais diretos, rivaroxabana, tromboembolismo venoso, obesidade mórbida, embolia pulmonar aguda

## Abstract

Direct oral anticoagulants (DOACs) have become the standard of care for acute and long-term therapy for venous thromboembolism (VTE) due to their efficacy and safety profiles. The 2021 International Society on Thrombosis and Haemostasis guidelines recommend using standard DOAC dosages in patients with BMI >40 kg/m^2^ or weight >120 kg. Use of DOACs remains uncertain in morbidly obese patients with VTE, including acute PE. A morbidly obese woman in her 30s who presented with acute worsening of dyspnea was diagnosed with acute intermediate-high risk acute pulmonary embolism and concomitant proximal deep vein thrombosis, constituting a clinically challenging scenario for treating her with rivaroxaban. Standard doses of rivaroxaban for acute and extended phase treatment of venous thromboembolism in individuals with morbid obesity at BMI>70 kg/m^2^ may be effective, and safe.

## Resumo

Devido à sua eficácia e aos seus perfis de segurança, os anticoagulantes orais diretos (DOACs) tornaram-se o padrão de cuidado para a terapia aguda e de longo prazo de tromboembolismo venoso (TEV). As diretrizes da Sociedade Internacional de Trombose e Hemostasia de 2021 recomendam o uso de dosagens padrão de DOACs em pacientes com índice de massa corporal (IMC) > 40 kg/m^2^ ou peso > 120 kg. O uso de DOACs em pacientes com obesidade mórbida e TEV, incluindo embolia pulmonar aguda, ainda não foi esclarecido. Uma mulher com obesidade mórbida na faixa dos 30 anos que apresentou piora aguda da dispneia foi diagnosticada com embolia pulmonar aguda de risco intermediário-alto e trombose venosa profunda proximal concomitante, com o cenário clínico desafiador de tratá-la com rivaroxabana. Doses padrão de rivaroxabana para tratamento e recorrência de tromboembolismo venoso em indivíduos com obesidade mórbida e IMC > 70 kg/m^2^ podem ser eficazes e seguras.

## INTRODUCTION

Systemic oral anticoagulation represents the cornerstone for both acute and long-term extended phase therapy of venous thromboembolism (VTE).^1^ Direct oral anticoagulants (DOACs) have become the standard of care, mostly due to their ease of use, efficacy, and safety profiles. Obesity remains a significant risk factor for VTE and there is a 9.2% prevalence of morbid obesity (body mass index [BMI] >40 kg/m^2^) in the United States, with an estimated 6.2-fold increased risk of VTE in obese Americans.^2^ The 2021 International Society on Thrombosis and Haemostasis (ISTH) guidelines recommend using standard dosages of DOACs in patients with BMI >40 kg/m^2^ or weight >120 kg.^1,3^ Despite these recommendations, their use in morbidly obese individuals remains uncertain. We present the case of an extremely obese patient (BMI >70 kg/m^2^) with acute intermediate-high risk pulmonary embolism (PE) and lower extremity deep vein thrombosis (DVT) successfully treated with rivaroxaban with demonstrable therapeutic anti-Xa levels throughout the acute phase of treatment.

## PART I: CLINICAL CASE

A morbidly obese woman in her 30s with a history of menorrhagia presented to the hospital with progressively worsening dyspnea on exertion. She weighed 243kg with a BMI of 72.3 kg/m^2^, was found to be hypoxemic, saturating 60% on room air, but otherwise hemodynamically stable. Upon initial evaluation, a computed tomographic angiography of the chest revealed an intermediate-to-high risk PE with right ventricular strain and elevated cardiac biomarkers. A concomitant proximal DVT of the left common femoral to distal peroneal veins was also noted on lower extremities Doppler ultrasound study.

## PART II: WHAT WAS DONE

She was promptly started on anticoagulation with intravenous unfractionated heparin and after 4 days was transitioned to the standard DVT/PE regimen of oral rivaroxaban 15 mg twice daily (BID) with plans to switch to rivaroxaban 20 mg daily maintenance dosing after 17 days of therapy. Ten days after starting rivaroxaban, she developed heavy menstrual bleeding (HMB). Although she remained hemodynamically stable with no need for transfusions, her persistent menstrual bleeding led to an earlier transition to 20 mg daily maintenance dose rivaroxaban after just 13 days on rivaroxaban 15 mg BID.

**Figure 1:**
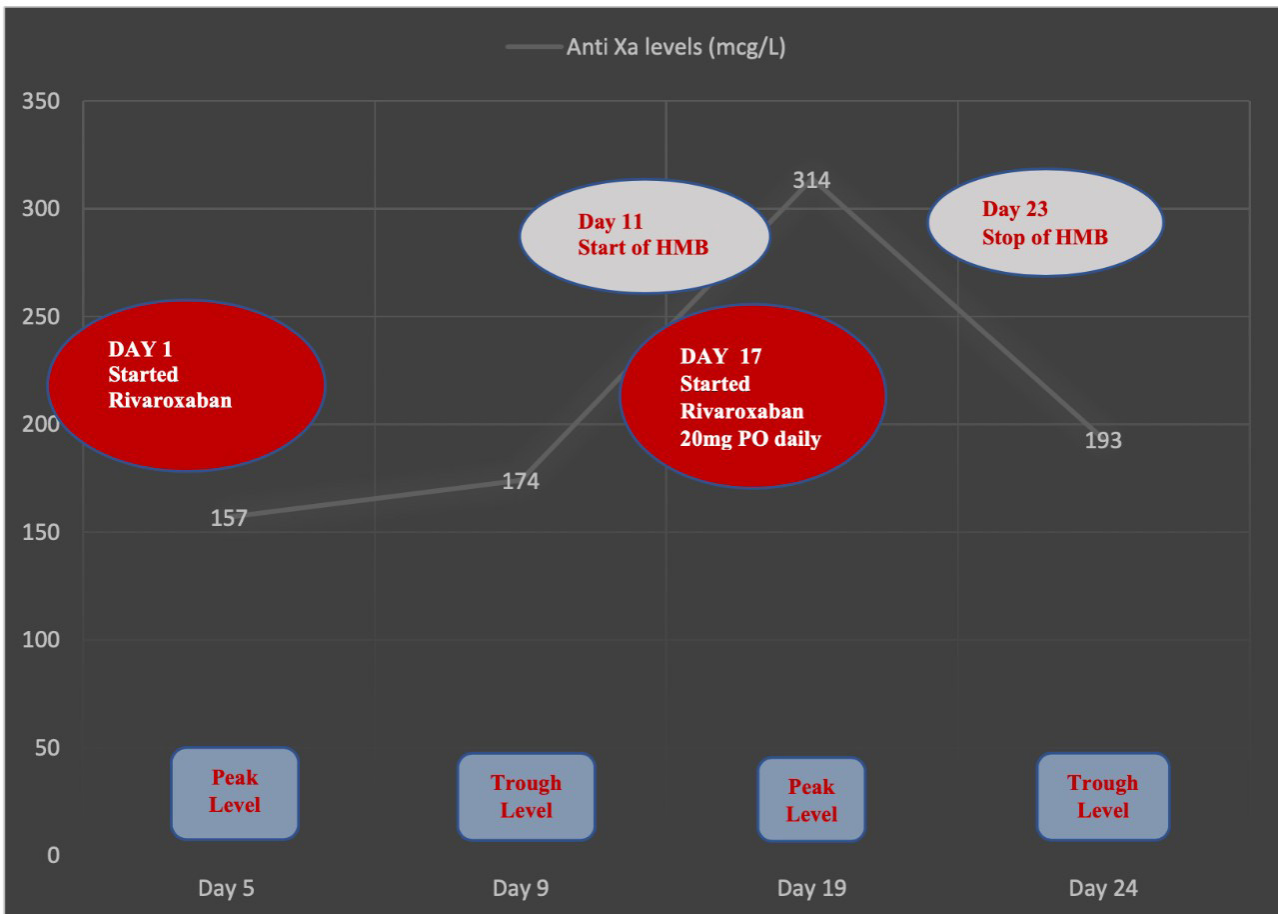
Peak and trough anti-Xa levels measured* on random days throughout patient’s hospital stay showed therapeutic levels were achieved in our extremely obese patient with a BMI of 72.3 kg/m^2^ using standard dosing of rivaroxaban for VTE. The highest Anti Xa level of 314 mcg/L was reported on day 19, correlating with worsening of the patient’s menorrhagia. Despite an early transition to rivaroxaban maintenance dosing of 20 mg daily, therapeutic anti-Xa levels were maintained, there was an improvement in menstrual bleeding, and there were no signs or symptoms of VTE recurrence.

Peak and trough anti-factor Xa levels (samples collected approximately 2-4 hours after and before the dose, respectively) were measured on days 5, 9, 19, and 24 of rivaroxaban therapy to assess anticoagulation efficacy (See Figure 1). Therapeutic anti-Xa levels were achieved and maintained throughout rivaroxaban anticoagulation, with the highest peak levels of 314 mcg/L (reference range: 182-408 mcg/L) seen 19 days into therapy.

*Measurements were performed with Quest diagnostic laboratories modified chromogenic anti-Xa assays which compare the optical density of the patient’s sample to calibrated rivaroxaban controls, correlating directly to plasma rivaroxaban concentrations. Reference ranges: Rivaroxaban 20 mg daily dose {peak (182-408 mcg/L), trough (3-153 mcg/L)}; rivaroxaban 15 mg daily dose {peak (180 - 408 mcg/L), trough (2-161 mcg/L)}.

With the onset of HMB, the switch to rivaroxaban 20 mg PO daily helped abate the patient’s risk of bleeding, which completely stopped after she was started on medroxyprogesterone 10 mg PO BID. Despite the switch to rivaroxaban 20 mg daily, there was no effect on efficacy as therapeutic trough anti-Xa levels of 193 mcg/L (reference range: 3 - 153 mcg/L) were still measured at discharge (day 24). Additionally, the patient had no further complications and did not develop any signs or symptoms of VTE recurrence. There was a significant improvement in her oxygenation and functional status and she was discharged home on the rivaroxaban 20 mg PO daily regimen for at least six months, with further clinical reassessment to decide if indefinite oral anticoagulation would be warranted.

## DISCUSSION

Although no clear dose adjustments for weight or BMI were stipulated for DOAC use in VTE treatment upon approval, their use in patients with BMI > 40 kg/m^2^ remains a challenge. Two pivotal trials, the *EINSTEIN-DVT* and *EINSTEIN-PE* trials, as well as a post-hoc analysis of their data, showed no significant difference in VTE recurrence and major bleeding among patients treated with rivaroxaban compared to those who received oral vitamin K antagonist across BMIs and body weights of 120-140 kg.^4-6^ This has since been replicated in observational and systemic reviews/metanalyses that are now showing that the DOACs are a safe and effective anticoagulation option for treatment of VTE patients with BMI > 40 kg/m^2^ or weight > 120 kg.^7-10^

Given that our patient was severely obese, we favored starting her on standard dose rivaroxaban over warfarin during the acute phase of her DVT/PE treatment coupled with close monitoring of her anti-Xa levels to ensure therapeutic drug levels were achieved and maintained.

Although the updated 2021 ISTH guidance statement suggests that use of standard doses of DOACs is appropriate for treatment of VTE regardless of BMI or weight, the recommendation for DOAC use in patients at extremes of weight is unclear, mainly owing to the paucity of prospective randomized clinical trial data to fully evaluate DOACs against other anticoagulation options in the extremely obese.^1^

Lachant et al. performed an observational cohort study in 107 patients, (morbidly obese patients, defined by BMI > 40 kg/m^2^; n=32 pts) who had intermediate-high risk acute PE after 3 months at the pulmonary vascular disorders clinic.^11^ Their group found no recurrent VTE events after 12 months of initial diagnosis and no significant difference in the rate of residual unmatched perfusion defects by lung scanning in the subset that took DOACs versus VKAs (47% vs. 50%) and there was no difference in the rate of chronic thromboembolic pulmonary hypertension (5% DOACs vs. 8% with VKAs). They concluded that DOACs appear to be safe and effective in morbidly obese patients with intermediate-high risk acute PE.

There is no doubt that uncertainty about efficacy and safety concerns continue to fuel clinician inertia on DOAC use in extremely obese patients. As a compromise, some clinicians find comfort in ensuring therapeutic drug levels to guide continual use in the extended phase of VTE therapy, as was the case with our patient. Our patient achieved and maintained expected therapeutic anti-Xa levels with both rivaroxaban 15 mg BID and 20mg daily doses. She did, however, develop HMB with the higher dose of rivaroxaban 15 mg twice daily, which was considered clinically relevant non-major bleeding (CRNMB), but improved with a dose reduction to 20 mg daily and bleeding completely stopped upon starting her on medroxyprogesterone contraception. Interestingly, this dose reduction did not affect the anti-Xa levels, which remained at therapeutic levels, nor was there a compromise in efficacy, as there were no signs or symptoms of VTE recurrence nor rebleeding. HMB is not uncommon with DOACs, especially rivaroxaban. Dose reductions, switching to another DOAC like apixaban, and initiation of hormonal contraception are safe options to curtail menstrual bleeding in anticoagulated patients.

Despite the initial ISTH guidance favoring DOAC-specific peak and trough monitoring of anti-Xa levels in patients with BMI > 40 kg/m^2^ or weight >120 kg and continuing the DOAC if levels are within expected therapeutic ranges as opposed to switching to warfarin, their 2021 update suggests against monitoring drug levels in these patients.^1^

Our case suggests that even in patients with BMI >70 kg/m^2^, therapeutic anti-Xa levels can be achieved with DOACs. Katel et al.^8^ evaluated five studies and found that DOACs had the same rate of recurrent venous thromboembolism risk and major bleeding events as other anticoagulants (VKAs) in severely obese patients. Similarly, Elshafei et al.^9^ reported that DOACs were non-inferior to warfarin in the severely obese.

Nevertheless, there is still concern regarding DOAC use in patients at extremes of weight, such as our patient, given the limited clinical outcome data. As a result, we side with Pandey et al.^12^ in saying that for patients with weight > 120 kg, standard doses of DOACs remain a safe and efficacious option over warfarin and for the extremely morbid obese with BMI>70 kg/m^2^, routinely checking DOAC-specific drug levels at least in the acute phase of VTE treatment should be considered to guide choice of anticoagulation for continuation of treatment in the extended phase. We believe that in 2023, weight should not be a deciding factor when choosing which oral anticoagulant to utilize for the treatment of VTE.

## CONCLUSIONS

Our case suggests that standard doses of rivaroxaban for the treatment of DVT/PE in individuals with morbid obesity with BMI > 70 kg/m^2^ can be effective and safe for acute and extended phase treatment of VTE. It also illustrates that increased dosages attempting to account for high bodyweight/fat can potentially result in major bleeding events. Despite the 2021 ISTH guidelines, further prospective studies and randomized controlled trials are warranted to fully assess the safety and efficacy of DOACs in VTE, particularly in challenging clinical scenarios involving extremely morbidly obese patients with intermediate-high risk PE. We acknowledge the limitations of use of DOAC blood levels as a surrogate for clinical outcomes in the treatment of VTE patients and the lack of standardized therapeutic targets as well as patient-specific or other factors that might affect accurate drug level measurements. Nevertheless, achieving expected range drug levels can be very reassuring in managing extremely morbidly obese VTE patients with DOACs. Additional studies will be essential to evaluate the need for universal and external validation of DOAC-specific anti-Xa levels and therapeutic targets for use in this challenging subpopulation of patients.
